# Human T-Lymphotropic Virus Type 1 (HTLV-1) and Regulatory T Cells in HTLV-1-Associated Neuroinflammatory Disease

**DOI:** 10.3390/v3091532

**Published:** 2011-08-25

**Authors:** Natsumi Araya, Tomoo Sato, Naoko Yagishita, Hitoshi Ando, Atae Utsunomiya, Steven Jacobson, Yoshihisa Yamano

**Affiliations:** 1 Department of Rare Diseases Research, Institute of Medical Science, School of Medicine, St. Marianna University, Kawasaki 216-8511, Japan; E-Mails: araya@marianna-u.ac.jp (N.A.); tomoo@marianna-u.ac.jp (T.S.); yagi@marianna-u.ac.jp (N.Y.); hando@marianna-u.ac.jp (H.A.); 2 Department of Hematology, Imamura Bun-in Hospital, Kagoshima 890-0064, Japan; E-Mail: autsunomiya@jiaikai.jp; 3 Viral Immunology Section, Neuroimmunology Branch, National Institute of Neurological Disorders and Stroke, National Institutes of Health, Bethesda, MD 20892, USA; E-Mail: jacobsons@ninds.nih.gov

**Keywords:** HTLV-1, HAM/TSP, ATL, CD4^+^CD25^+^CCR4^+^ T cell, regulatory T cell, exFoxp3^+^ cell, inflammation, immune-dysfunction

## Abstract

Human T-lymphotropic virus type 1 (HTLV-1) is a retrovirus that is the causative agent of adult T cell leukemia/lymphoma (ATL) and associated with multiorgan inflammatory disorders, including HTLV-1-associated myelopathy/tropical spastic paraparesis (HAM/TSP) and uveitis. HTLV-1-infected T cells have been hypothesized to contribute to the development of these disorders, although the precise mechanisms are not well understood. HTLV-1 primarily infects CD4^+^ T helper (Th) cells that play a central role in adaptive immune responses. Based on their functions, patterns of cytokine secretion, and expression of specific transcription factors and chemokine receptors, Th cells that are differentiated from naïve CD4^+^ T cells are classified into four major lineages: Th1, Th2, Th17, and T regulatory (Treg) cells. The CD4^+^CD25^+^CCR4^+^ T cell population, which consists primarily of suppressive T cell subsets, such as the Treg and Th2 subsets in healthy individuals, is the predominant viral reservoir of HTLV-1 in both ATL and HAM/TSP patients. Interestingly, CD4^+^CD25^+^CCR4^+^ T cells become Th1-like cells in HAM/TSP patients, as evidenced by their overproduction of IFN-γ, suggesting that HTLV-1 may intracellularly induce T cell plasticity from Treg to IFN-γ^+^ T cells. This review examines the recent research into the association between HTLV-1 and Treg cells that has greatly enhanced understanding of the pathogenic mechanisms underlying immune dysregulation in HTLV-1-associated neuroinflammatory disease.

## 1. Introduction

Human T-lymphotropic virus type 1 (HTLV-1) is a retrovirus associated with chronic, persistent infection of human T cells. HTLV-1 infection is endemic in Japan, the Caribbean, and part of South America, Africa, the Middle East, and Melanesia [1]. Studies conducted in HTLV-1 endemic areas have demonstrated that HTLV-1 infection is associated with a variety of human diseases, including an aggressive mature T cell malignancy termed adult T-cell leukemia (ATL) [2], which is defined as neoplastic growth of HTLV-1-infected T cells. HTLV-1 is also associated with non-neoplastic inflammatory conditions such as HTLV-1-associated myelopathy/tropical spastic paraparesis (HAM/TSP) [3,4], uveitis [5], Sjögren syndrome [6], bronchoalveolitis, arthritis [7], and polymyositis [8], where high tissue concentrations of HTLV-1 infected T lymphocytes have been observed. Importantly, some patients have more than one of these HTLV-1-associated inflammatory conditions [9].

Although HTLV-1-associated disorders have been extensively studied, the exact mechanism by which HTLV-1 induces these inflammatory conditions is not completely understood. The proviral load of HTLV-1 may contribute to development of HTLV-1-associated inflammatory conditions, since the number of HTLV-1-infected T cells circulating in the peripheral blood is higher in patients with HAM/TSP than in asymptomatic HTLV-1-infected individuals [10,11], and is even higher in the cerebrospinal fluid of patients with HAM/TSP [12]. In HAM/TSP patients, the proviral load correlates with not only the percentage of activated CD4^+^ T cells but also with that of HTLV-1-specific CD8^+^ cytotoxic T lymphocytes (CTLs) [11,13]. These HTLV-1-specific CTLs produce various cytokines, such as IFN-γ and TNF-α, that may suppress viral replication and kill infected cells and/or promote bystander activation and killing of nearby resident cells in the central nervous system (CNS) [14–17]. In addition, increased viral expression, particularly of the transactivating viral gene encoding HTLV-1 Tax, has also been hypothesized to play a role in HTLV-1 disease progression [11,12]. Transgenic mice expressing HTLV-1 Tax develop an inflammatory arthropathy [18], and transgenic rats expressing HTLV-1 env-pX develop destructive arthropathy, Sjögren syndrome, vasculitis, and polymyositis [19]. These findings support the hypothesis that HTLV-1 *tax* is one of the exogenous retrovirus genes responsible for immune dysregulation.

HTLV-1 Tax is a transactivator/oncoprotein that has potent effects on infected T cells, including activation of nuclear factor(NF)-κB [20] with subsequent enhancement of cell activation and proliferation and expression of various cellular genes, such as IL-2 [21], the α-chain of the IL-2 receptor (IL-2Rα) [22], IL-15 [23], and IL-15Rα [24]. Such virus-induced intracellular activation may directly contributes to T cell activation and the *ex vivo* T cell proliferation observed in patients with HAM/TSP [25]. These findings suggest that invasion by HTLV-1-infected T cells, together with viral gene expression and cellular-signaling mechanisms, trigger a strong virus-specific immune response and increased proinflammatory cytokine production, leading to CNS inflammation and autologous tissue damage. However, the precise mechanisms underlying the induction of immune activation by HTLV-1-infected T cells are not well understood.

## 2. HTLV-1 and Regulatory T Cells

The recent discovery of regulatory T cells (Treg cells) has generated new opportunities for and increased interest in elucidating the above mentioned mechanisms. In healthy individuals, the Treg cells, a subset of CD4^+^CD25^+^ T cells, play a key role in maintaining immune system homeostasis by suppressing the proliferation of and cytokine production by pathogenic T cells [26]. Although Treg cells are phenotypically similar to activated T cells, they can be identified *ex vivo* by their intracellular expression of the transcriptional regulator Foxp3 [27], which is critical in the development and functioning of Treg cells in both mice and humans. Significant reductions in Foxp3 expression and/or Treg cell function have been observed in patients with several types of human autoimmune diseases [28], suggesting that defects in Foxp3 expression and/or Treg functioning may precipitate loss of immunological tolerance. CD4^+^CD25^+^ T cells are also the predominant viral reservoir in the peripheral blood of HTLV-1-infected individuals [29]. Recently, significant reductions in Foxp3 expression and Treg cell function have been observed in CD4^+^CD25^+^ T cells from patients with HAM/TSP [30–34]. Furthermore, decreased expression levels of CTL antigen-4 (CTLA-4), a Treg-associated immune-suppressive molecule, and glucocorticoid-induced tumor necrosis factor receptor-related protein (GITR) have also been observed on the CD4^+^CD25^+^ T cells of HAM/TSP patients [30,34]. Notably, overexpression of HTLV-1 Tax has been observed to reduce Foxp3 expression and inhibit the suppressive function of Treg cells *in vitro* [30]. Furthermore, because of a Tax-induced defect in TGF-β signaling, Foxp3 expression was decreased and Treg functions were impaired in patients with HAM/TSP [35]. Recently, significantly decreased numbers of CD4^+^CD25^+^Foxp3^+^ Treg cells were observed in transgenic mice expressing HTLV-1 Tax that develop an inflammatory arthropathy [36]. In addition, increased viral expression of the HTLV-1 bZIP factor (*HBZ*) gene encoding the minus strand of HTLV-1 has also been suggested to play a role in HTLV-1 disease progression [37], and CD4^+^Foxp3^+^ Treg cells in HBZ transgenic mice were functionally impaired [38]. These findings indicate that HTLV-1-induced dysfunctioning of CD4^+^CD25^+^ Treg cells may be one of the mechanisms underlying the induction of immune activation by HTLV-1-infected T cells.

In contrast to the decreased expression of Foxp3 in CD4^+^CD25^+^ T cells observed in HAM/TSP patients [30–34], most CD4^+^CD25^+^ ATL cells have been shown to express Foxp3 in patients with ATL [39,40]. Therefore, it has been hypothesized that ATL cells may be derived from Treg cells [41]. Interestingly, some ATL cells exhibit immunosuppressive functions similar to those of Treg cells, which may contribute to clinically observed cellular immunodeficiency in ATL patients [41–43], although some of these ATL cells lose this regulatory function [44].

## 3. HTLV-1 and CD4^+^CD25^+^CCR4^+^ T Cells

**Figure 1.**
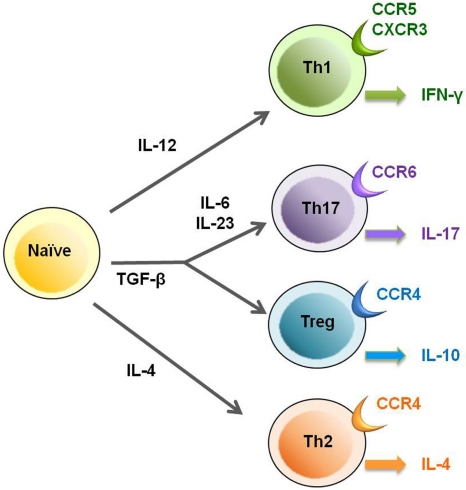
T cell subsets of CD4^+^ T helper cells. Th cells are differentiated from naïve CD4^+^ T cells into 4 major lineages: Th1, Th2, Th17, and T-regulatory (Treg) cells. Each Th subset exhibits characteristic functions, patterns of cytokine secretion, and expression of specific chemokine receptors.

Although HTLV-1 has been reported to infect a number of cell types both *in vitro* and *in vivo* [29,45–49], CD4^+^ Th cells, which play a central role in adaptive immune responses, are the predominant viral reservoir in the peripheral blood [50]. To understand the effects of HTLV-1 infection on the functioning of CD4^+^ Th cells, it is necessary to discover if, and if so which of the Th subpopulations is preferentially infected with HTLV-1. Based on their functions, patterns of cytokine secretion, and expression of specific transcription factors and chemokine receptors, CD4^+^ Th cells, which are differentiated from naïve CD4^+^ T cells, are classified into four major lineages: Th1, Th2, Th17, and Treg cells (Figure 1).

The chemokine receptor CCR4 has recently been found to be expressed on HTLV-1-infected leukemia cells in ATL patients [51]. Because CCR4 is known to be selectively expressed on Treg and Th2 cells [51–53] (Figure 1) and because most ATL cells express high levels of Foxp3, it has been hypothesized that ATL cells may be derived from Treg cells [41]. Although it has been demonstrated that CD4^+^CD25^+^ T cells in HAM/TSP patients exhibit reduced Foxp3 expression and Treg suppression [30–33] and that HTLV-1-infected CD4^+^ T cells in HAM/TSP patients produce Th1 cytokines (IFN-γ) [16,30], it has also been observed that CCR4 selectively overexpresses on HTLV-1-infected T cells in HAM/TSP patients [54]. Furthermore, the majority of CD4^+^CD25^+^CCR4^+^ T cells have been found to be infected with HTLV-1 and this T cell subset has increased numbers in HAM/TSP patients [54]. Thus, CD4^+^CD25^+^CCR4^+^ T cells are a major reservoir of HTLV-1-infected T cells, which are increased in numbers in both HAM/TSP and ATL patients.

## 4. HTLV-1 and Foxp3^−^CD4^+^CD25^+^CCR4^+^ T Cells

**Figure 2.**
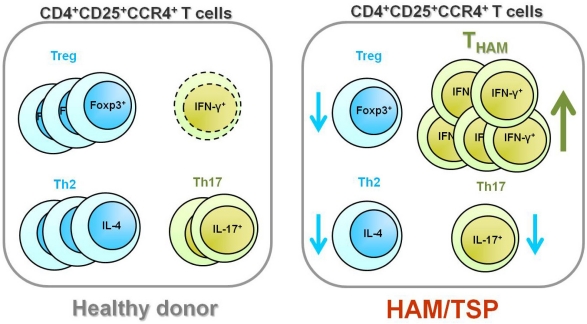
Cellular components of CD4^+^CD25^+^CCR4^+^ T cells in healthy donors and HAM/TSP patients. In healthy donors, the CD4^+^CD25^+^CCR4^+^ T cell population primarily consists of suppressive T cell subsets, such as Treg and Th2, whereas that of HTLV-1-associated myelopathy/tropical spastic paraparesis (HAM/TSP) patients consists of an increased number of IFN-γ-producing Foxp3^−^CD4^+^CD25^+^CCR4^+^ T cells (T_HAM_ cells).

Although CCR4 is known to be selectively expressed on Treg and Th2 cells in healthy individuals, more detailed flow cytometric analysis of Foxp3 expression in CD4^+^CD25^+^CCR4^+^ T cells of HAM/TSP patients demonstrated that the frequency of the Foxp3^−^ population was greatly increased in CD4^+^CD25^+^CCR4^+^ T cells [54]. Moreover, analysis of proinflammatory cytokine expression in this Foxp3^−^CD4^+^CD25^+^CCR4^+^ T cell subset demonstrated that these cells uniquely produced multiple proinflammatory cytokines such as IL-2, IL-17, and few IFN-γ in healthy individuals while Foxp3^+^CD4^+^CD25^+^CCR4^+^ T cells (Treg cells) did not. Furthermore, it was demonstrated that HAM/TSP patients had only few Foxp3^+^CD4^+^CD25^+^CCR4^+^ T cells that did not produce such cytokines [54]. The Foxp3^−^CD4^+^CD25^+^CCR4^+^ T cells in HAM/TSP were greater in number and overproduced IFN-γ [54]. Further, the proportion of these IFN-γ-producing Foxp3^−^CD4^+^CD25^+^CCR4^+^ T cells may have a functional consequence, since the presence of this subpopulation could be correlated with disease activity and severity of HAM/TSP *in vivo* [54]. Thus, in a CD4^+^CD25^+^CCR4^+^ T cell population that mainly consists of suppressive T cell subsets such as Treg and Th2 under healthy conditions, IFN-γ-producing Foxp3^−^CD4^+^CD25^+^CCR4^+^ T cells, rarely encountered in healthy individuals, were increased in number and overproduced IFN-γ in HAM/TSP patients (Figure 2). We therefore propose to call this IFN-γ^+^Foxp3^−^CD4^+^CD25^+^CCR4^+^ T cell subset T_HAM_ cells. Interestingly, increased numbers of Foxp3^low^CD4^+^CD25^+^ memory T cells, which have cytokine secretion patterns similar to those of T_HAM_ cells, have recently been observed in patients with active systemic lupus erythematosus (SLE) [55]. Therefore, it would be of interest to build on this finding by confirming whether this newly defined unique T cell subset, which has been observed in both HAM/TSP and SLE patients, is found in both these patient groups and can be functionally deregulated in other immunological diseases.

**Figure 3.**
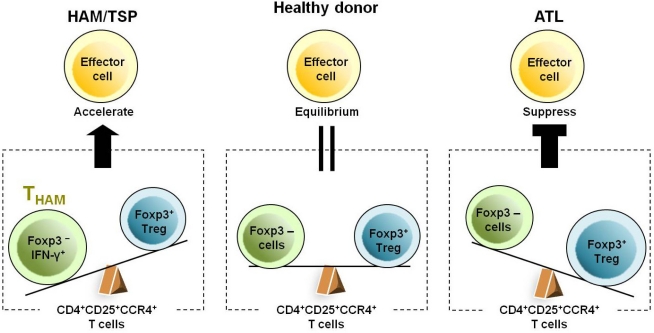
Differential immune responses and T_HAM_/Treg ratios in CD4^+^CD25^+^CCR4^+^ T cells in HAM/TSP and adult T cell leukemia/lymphoma (ATL) patients.

Although most CD4^+^CD25^+^CCR4^+^ T cells are infected with HTLV-1 in both HAM/TSP and ATL patients [54,56], the ratio of T_HAM_ cells (CCR4^+^Foxp3^−^ with IFN-γ production) to Treg cells (CCR4^+^Foxp3^+^ with no cytokine production) in the CD4^+^CD25^+^CCR4^+^ T cell subset has been found to be high in HAM/TSP patients but low in ATL patients [54]. This differential T_HAM_/Treg ratio in HTLV-1-infected T cells may be associated with the differential immune responses observed between HAM/TSP and ATL patients (Figure 3). ATL patients tend to have very low numbers of Tax-specific CD8^+^ T cells in peripheral blood mononuclear cells (PBMCs) and to develop opportunistic infections [57,58], while HAM/TSP patients tend to have high numbers of Tax-specific CD8^+^ CTLs [11,12,14,59]. As CD4^+^CD25^+^ T cells with high levels of Foxp3 expression have been reported to have an immunosuppressive function in ATL patients [41–43], the increased number of CD4^+^CD25^+^CCR4^+^ leukemia T cells with Treg functions observed in ATL patients may contribute to their clinically observed cellular immunodeficiency. However, HAM/TSP patients show very high cellular and humoral immune responses, such as high proportions of Tax-specific CD8^+^ T cells, as well as cytomegalovirus (CMV)-specific CD8^+^ T cells in the PBMCs [14,33]; high antibody titer to HTLV-1 [9]; and increased production of proinflammatory cytokines, such as IL-12 and IFN-γ [60]. It has been reported that CD4^+^CD25^+^ T cells with low expression of Foxp3 [30] and HTLV-1 Tax-expressing Foxp3^+^ Treg cells [61] extracted from HAM/TSP patients exhibit defective immunosuppressive functioning. Moreover, it has been demonstrated that HTLV-1-infected IFN-γ-overproducing CD4^+^CD25^+^CCR4^+^Foxp3^−^ T cells (T_HAM_ cells) increase in number in HAM/TSP patients, and their levels can be correlated with disease severity [54]. Thus, CD4^+^CD25^+^CCR4^+^ T cells with increased proinflammatory functioning, together with a defective Treg compartment [30–33,54], may overcome the regulatory effect of HTLV-1-uninfected Treg cells [61] and at least partly account for the heightened immune response observed in HAM/TSP patients. Collectively, these observations support the hypothesis that an imbalance in the T_HAM_/Treg ratio in HTLV-1-infected CD4^+^CD25^+^CCR4^+^ T cells is an important contributing factor in the immunological differences in host immune response observed between HAM/TSP and ATL patients (Figure 3).

## 5. Increased Numbers of CD4^+^Foxp3^+^ Cells in HAM/TSP Patients

**Figure 4.**
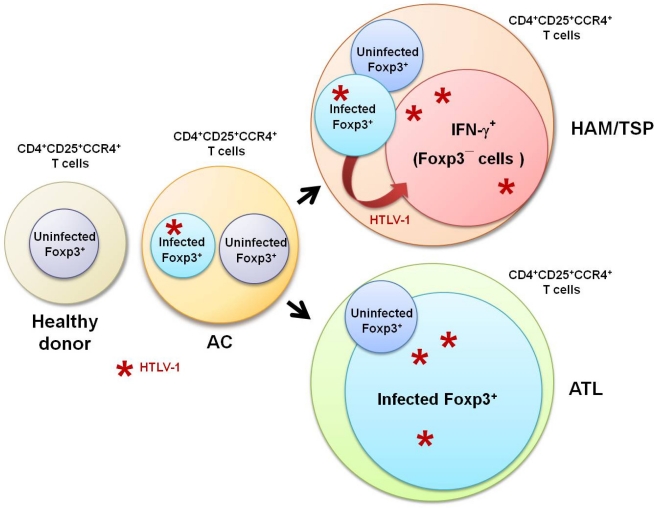
Scheme of proportion of each cellular component in CD4^+^CD25^+^CCR4^+^ T cells of healthy donors, asymptomatic carriers (AC), and patients with HAM/TSP or ATL. Although the proportion of Foxp3^+^ cells among the CD4^+^CD25^+^CCR4^+^ T cells is lower in HAM/TSP patients, the overall number of CD4^+^Foxp3^+^ cells in HAM/TSP patients is higher than that in healthy donors. In ATL patients, the majority of CD4^+^CD25^+^CCR4^+^ T cells are Foxp3^+^ cells.

Recently, it has been reported that the number of CD4^+^Foxp3^+^ cells increases in HTLV-1-infected asymptomatic carriers, and is even higher in patients with HAM/TSP [61]. Although this report initially appears to conflict with the observations described above, it may not. In contrast to the decreased number of CD4^+^ T cells in patients with human immunodeficiency virus (HIV) infection, the number of HTLV-1 infected CD4^+^ T cells—most of which are CD4^+^CD25^+^CCR4^+^ T cells—in HAM/TSP patients is greatly increased. Therefore, although the percentage of Foxp3^+^ cells among the CD4^+^CD25^+^CCR4^+^ T cells is lower, the overall number of CD4^+^Foxp3^+^ cells in HAM/TSP patients may be higher than that in healthy donors (Figure 4). Indeed, when we analyzed the number of Foxp3^+^ cells in healthy donors and HAM/TSP patients, we found it to be nearly equivalent between the two groups or slightly higher in HAM/TSP patients [54]. This difference (from slightly high to higher) would depend on the number of HTLV-1-infected CD4^+^ T cells in the samples tested. Importantly, Toulza *et al*. demonstrated that the rate of CTL-mediated lysis was negatively correlated with the number of HTLV-1-Tax^−^CD4^+^Foxp3^+^ cells, but not with the number of Tax^+^CD4^+^Foxp3^+^ cells [61], again suggesting that HTLV-1-infected Treg cells lose their regulatory function, while HTLV-1-uninfected Treg cells contribute substantially to immune control of HTLV-1 infection.

## 6. Does the T_HAM_ Cell Population Include exFoxp3^+^ Cells?

**Figure 5.**
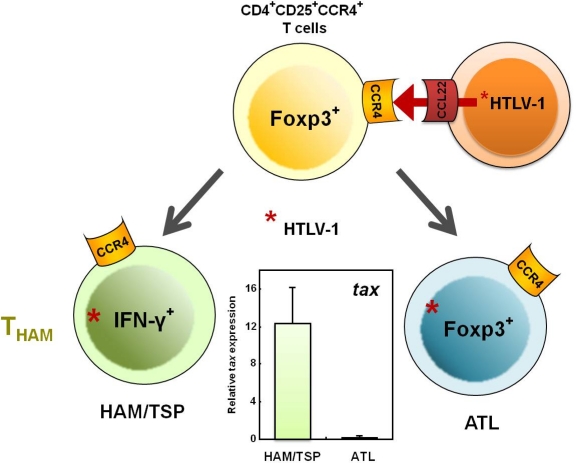
Differential fate of HTLV-1-infected CD4^+^CD25^+^CCR4^+^ T cells in HAM/TSP and ATL patients. After HTLV-1 infection, CD4^+^CD25^+^CCR4^+^ T cells in HAM/TSP patients, which are primarily Th2 and Treg cells before infection, become IFN-γ^+^Foxp3^−^ T cells (T_HAM_ cells) with high levels of intracellular HTLV-1 *tax* expression. In ATL patients, leukemogenesis develops and the Foxp3^+^ Treg phenotype is maintained.

According to Hieshima *et al*.’s recent delineation of the molecular mechanism underlying HTLV-1 tropism to CCR4^+^CD4^+^ T cells [60], HTLV-1 Tax does not induce expression of CCR4, but Tax does induce expression of CCL22, which is the ligand for CCR4. Therefore, HTLV-1-infected T cells produce CCL22 through Tax and selectively interact with CCR4^+^CD4^+^ T cells, resulting in preferential transmission of HTLV-1 to CCR4^+^CD4^+^ T cells (Figure 5). In HTLV-1-seronegative healthy individuals, CD4^+^CD25^+^CCR4^+^ T cell populations primarily consist of suppressive T cell subsets, such as Treg and Th2 cells [61]. However, as described above, cells of this T cell subset become Th1-like cells that overproduce IFN-γ in patients with HAM/TSP, while leukemogenesis develops and maintains the Foxp3^+^ Treg phenotype in ATL patients (Figure 5).

**Figure 6.**
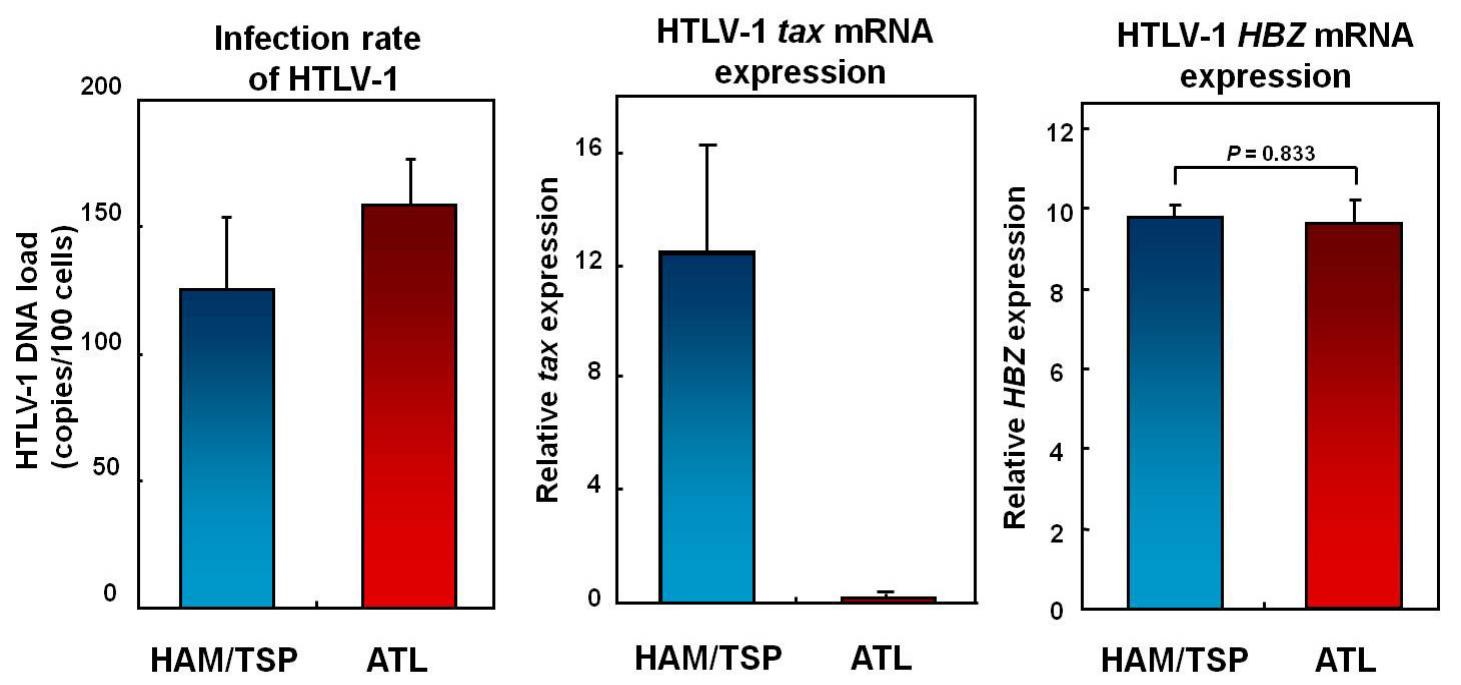
Increased HTLV-1 *tax* mRNA expression in CD4^+^CD25^+^CCR4^+^ T cells in HAM/TSP patients. The HTLV-1 proviral load in CD4^+^CD25^+^CCR4^+^ T cells from HAM/TSP and ATL patients was quantified by real-time PCR (left panel, n = 3). Expression levels of HTLV-1 *tax* mRNA (center panel, HAM/TSP: n = 4, ATL: n = 3) and *HBZ* mRNA (right panel, n = 5) in CD4^+^CD25^+^CCR4^+^ T cells from HAM/TSP and ATL patients were quantified by real-time RT-PCR. Data are presented as mean ± standard error.

To determine whether HTLV-1 expression contributes to the differential fate of HTLV-1-infected CD4^+^CD25^+^CCR4^+^ T cells between HAM/TSP and ATL patients, differences in the HTLV-1 proviral load and the HTLV-1 *tax* mRNA and HTLV-1 *HBZ* mRNA expression of these populations were analyzed (Figure 6). Although HTLV-1 *tax* mRNA expression in CD4^+^CD25^+^CCR4^+^ T cells was found to be significantly higher in HAM/TSP patients than in ATL patients, HTLV-1 proviral DNA loads and *HBZ* mRNA expression levels were found to be equivalent in the two groups [54] (Figure 6). This high HTLV-1 Tax expression in HAM/TSP CD4^+^CD25^+^CCR4^+^ T cells (Foxp3^−^) and low HTLV-1 Tax expression in ATL CD4^+^CD25^+^CCR4^+^ T cells (Foxp3^+^) suggests that intracellular HTLV-1 expression may act as a “switch” that directs T cell plasticity from Foxp3^+^ Treg cells to IFN-γ^+^Foxp3^−^ T cells. Indeed, a recent report highlighted that loss of Foxp3 in Treg cells and acquisition of IFN-γ may result in conversion of suppressor T cells into highly autoaggressive lymphocytes (exFoxp3^+^ cells), which can contribute to the development of autoimmune conditions [62,63]. These findings support the hypothesis that HTLV-1 *tax* may be one of the exogenous retrovirus genes responsible for immune dysregulation through its interference in the equilibrium between inflammation and tolerance. This hypothesis is currently being tested as a means of elucidating the precise molecular mechanisms by which HTLV-1 influences the fate and function of CD4^+^CD25^+^CCR4^+^ T cells, especially Foxp3^+^ Treg cells. Further research investigating this hypothesis using animal models is required, as is further work to pathologically identify the exFoxp3^+^ cells in the spinal cord lesions of HAM/TSP patients.

## 7. Mechanisms Underlying Increased HTLV-1 Tax Expression in HAM/TSP Patients

As described above, higher levels of HTLV-1 Tax expression have been observed in HAM/TSP patients [11], and a correlation between Tax expression and disease risk [64] has been identified. Both findings, together with experimental evidence [65] and theoretical justification [66] for selective proliferation of HTLV-1 expressing T cells *in vivo*, indicate that increased HTLV-1 provirus expression may play an important role in the pathogenesis of HAM/TSP. However, the molecular mechanisms underlying the increased levels of HTLV-1 provirus expression in HAM/TSP patients are not understood. Evidence continues to accumulate that the genomic integration site of HTLV-1 provirus affects the level of provirus expression. Continued accumulation of evidence is aided by the availability of the human genome sequence, which has enabled large-scale research into HTLV-1 integration sites. This research has demonstrated that the provirus integration sites of HTLV-1 *in vivo* are not randomly distributed within the human genome but rather associated with transcriptionally active regions [67,68]; that the frequent integration into these transcription units is associated with increased levels of provirus expression; and, importantly, that the increased number of integration sites in transcription units is associated with HAM/TSP [68]. Future research should endeavor to elucidate the mechanisms underlying the immune dysregulation observed in HAM/TSP patients.

## 8. Conclusion

HTLV-1 initiates persistent infection of CD4^+^ T cells and results in the development of HAM/TSP, a chronic neuroinflammatory disorder characterized by very high strong cellular and humoral immune responses. Because a higher viral load in HTLV-1-infected individuals increases the risk of HAM/TSP and is associated with high cellular and humoral immune responses, HTLV-1 infection-induced immune dysregulation may play an important role in the development and pathogenesis of this disease. The recent discovery of Treg cells has provided new opportunities for and generated increased interest in elucidating the mechanisms underlying the induction of immune activation by HTLV-1-infected T cells. Among the CD4^+^ T helper cell populations that play a central role in adaptive immune responses, the CD4^+^CD25^+^CCR4^+^ T cell population, which primarily consists of suppressive T cell subsets, such as the Treg and Th2 subsets, in healthy individuals, is the predominant viral reservoir of HTLV-1 in both ATL and HAM/TSP patients. Interestingly, cells of this T cell subset become Th1-like cells, overproducing IFN-γ in HAM/TSP patients, while leukemogenesis develops and maintains the Foxp3^+^ Treg phenotype in ATL patients. These results indicate that HTLV-1 may intracellularly induce T cell plasticity from Treg to IFN-γ^+^ T cells, which may contribute to the development of HAM/TSP. As such, these results support the hypothesis that HTLV-1 is one of the exogenous retrovirus genes responsible for immune dysregulation through its interference in the equilibrium maintained among host immune responses. Because the majority of immune disorders are of unknown etiology, the discovery of HTLV-1 and its association with inflammatory conditions has greatly enhanced our understanding of the pathogenic mechanisms underlying organ-specific immune disorders. Further investigation of the mechanism underlying HTLV-1 action in the immune system may result in identification of new molecular pathways that will further elucidate the basic mechanisms underlying immune-mediated disorders.
